# A combined nutritional index and mortality in patients with peritoneal dialysis

**DOI:** 10.1080/0886022X.2025.2541069

**Published:** 2025-08-10

**Authors:** Wenjun Lin, Juan Wu, Xiaojiang Zhan, Yueqiang Wen, Xiaoyang Wang, Xiaoran Feng, Fenfen Peng, Na Tian, Ning Su, Qingdong Xu, Niansong Wang, Xiangwei Liao, Xianfeng Wu

**Affiliations:** ^a^ Department of Nephrology, Shanghai Jiao Tong University Affiliated Sixth People’s Hospital, Shanghai, China; ^b^ Department of Nephrology, Zhejiang Provincial People’s Hospital, Hangzhou, China; ^c^ Department of Nephrology, The First Affiliated Hospital of Nanchang University, Nanchang, China; ^d^ Department of Nephrology, The Second Affiliated Hospital of Guangzhou Medical University, Guangzhou, China; ^e^ Department of Nephrology, The First Affiliated Hospital of Zhengzhou University, Zhengzhou, China; ^f^ Department of Nephrology, Jiujiang No. 1 People’s Hospital, Jiujiang, China; ^g^ Department of Nephrology, Zhujiang Hospital of Southern Medical University, Guangzhou, China; ^h^ Department of Nephrology, General Hospital of Ningxia Medical University, Yinchuan, China; ^i^ Department of Hematology, The Sixth Affiliated Hospital of Sun Yat-Sen University, Guangzhou, China; ^j^ Department of Nephrology, Jiangmen Central Hospital, Jiangmen, China; ^k^ Department of Gastroenterology, Shanghai Jiao Tong University Affiliated Sixth People’s Hospital, Shanghai, China; ^l^ Department of Nephrology, Shanghai Eighth People’s Hospital, Shanghai, China

**Keywords:** Total cholesterol/(body mass index × serum albumin, mortality, peritoneal dialysis, nutrition

## Abstract

**Background:**

A combination of multiple nutritional indexes may more comprehensively and favorably reflect dialysis patient’s prognosis. In the present study, we aimed to evaluated the association between total cholesterol/(body mass index × serum albumin) (TC/BA) and mortality in patients with continuous ambulatory peritoneal dialysis (CAPD).

**Methods:**

We conducted a multicenter retrospective cohort study that included 2907 incident Chinese CAPD patients from seven peritoneal dialysis centers in China between January 1, 2005, and May 31, 2023. The primary outcome was all-cause mortality. We used restricted-cubic-spline plots to explore the shape of the association between TC/BA and outcome. Cause-specific hazard models examined the association between TC/BA and mortality.

**Results:**

Of 2907 patients, 754 (25.9%) patients died, including 351 (46.6%) deaths due to cardiovascular disease. A positive linear relationship was observed between TC/BA and all-cause mortality (nonlinear, *p* = 0.374). In the multivariate cause-specific hazard model, a per-1.0 increase of TC/BA had a 1.29-fold (95% confidence interval [CI] 1.19–1.39) risk of all-cause mortality in the total population. Similar trends were observed in subgroup analyses (all P for interactions > 0.05). Notably, the hazard ratio (HR) of TC/BA (per-1.0 increase) was 1.32 (95% CI 1.23–1.41), 1.29 (95% CI 1.19–1.41), and 1.25 (95% CI 1.22–1.27) times higher than the HR of body mass index (per-1.0 decrease), total cholesterol (per-10 increase), and serum albumin (per-1.0 decrease), respectively.

**Conclusions:**

TC/BA was positively linearly related to mortality and may outperform each index in assessing CAPD patient prognosis. The combined nutritional index may more comprehensively and favorably evaluate CAPD patients’ prognosis.

## Introduction

Many patients are malnourished when they begin dialysis. Moderate to severe malnutrition is associated with an increased risk of death in patients on both hemodialysis and peritoneal dialysis (PD). Early publications estimated that 40–66% of patients on peritoneal dialysis in the United States are malnourished [1–6]. In a subsequent study that defined protein-energy wasting (PEW) by serum albumin of <3.8 g/dL, 63% of patients on peritoneal dialysis were classified as malnourished [7]. As an example, in one study, approximately 75% of patients initiating peritoneal dialysis were malnourished [8].

In 2008, the International Society for Renal Nutrition and Metabolism (ISRNM) published standardized criteria for the diagnosis: biochemical parameters (serum albumin, serum prealbumin, or total cholesterol), body mass index (BMI), lean body mass, and dietary intake [9]. It is often difficult to diagnose low muscle mass or muscle loss in clinical practice [10]. Currently, there are no clinically helpful, uniform, and reproducible measures of lost muscle mass and methods for assessing the presence of accelerated muscle protein catabolism [11]. Daily dietary protein intake logs can also estimate the patient’s nutritional status. Low dietary protein levels, as assessed by three-day dietary records, are associated with increased mortality in patients on peritoneal dialysis [12]. However, it is challenging to determine protein intake using dietary logs because extensive food record keeping by the patient is required, and a dietitian trained explicitly in evaluating food intake histories is required. Other biochemical markers are associated with malnutrition and/or adverse outcomes but are not commonly measured. For example, lower levels of serum thymosin B4 are associated with lower serum albumin levels, lower subjective global nutritional assessment scores, higher levels of inflammatory markers such as serum C reactive protein, and indicators of atherosclerosis such as increased common carotid artery intimal thickness [13]. In clinical practice, serum albumin, total cholesterol, and BMI, which are convenient and easy to measure, are more likely to be promoted and used for nutritional status in dialysis patients. Notably, using different nutritional indicators to define malnutrition can show different nutritional status. A combination of multiple indicators more comprehensively reflects the patient’s nutritional status. A recent study combined serum albumin and total cholesterol to evaluate the association between nutritional status and prognosis in patients with PD. We found that albumin to total cholesterol ratio before the start of PD between 0.77 and 0.82 was associated with a lower risk of death than a higher or lower ratio, resulting in a U-curve association [14]. In addition, a study published by The Lancet in 2009 involving 900,000 adults showed that overweight and underweight people both had a mortality rate higher than normal-weight people as defined by BMI [15]. As an important nutrition index, BMI was not included in the study [14]. A combination of three indicators more comprehensively reflects patients’ nutritional status. Therefore, we constructed a combined nutritional index, total cholesterol/(BMI × serum albumin), to more comprehensively present patients’ nutritional status. We then evaluated the association between the combined nutritional index and mortality in patients with continuous ambulatory peritoneal dialysis (CAPD). In addition, we wondered whether the combined index outperformed each index in assessing CAPD patients’ prognosis.

## Methods

### Study design and participants

We conducted a multicenter retrospective cohort study that included 5128 incident Chinese CAPD patients from seven PD centers in China between January 1, 2005, and May 31, 2023. Patients < 18 years old or follow-up time < 3 months were excluded. In addition, severe liver disease affects albumin synthesis, lowering lipid drugs affect total cholesterol, diuretics affect BMI and lipid metabolism, and glucocorticoids affect lipid metabolism. Thus, patients with severe liver disease, lowering lipid drugs, diuretics, edema, or glucocorticoids were excluded accordingly. The data were anonymous, and the need for informed consent was waived. This study was approved by the local institutional ethics committee (jjsdyrmyy-yxyj-2021-107), which waived the need for written informed consent because this study was an observational study of case data, and no privacy issues were involved.

### Data collection and follow-up

Two well-trained nurses collected demographic data, comorbidities, and laboratory data one week (5.4 ± 1.1 days) before the start of PD in each facility, including age at study entry, sex, BMI, current smoker, current alcohol use, comorbidities (diabetes mellitus, prior cardiovascular disease [CVD], and hypertension), medication use (calcium channel blockers [CCB], *β*-blockers, *α*-blockers, and angiotensin-converting enzyme inhibitors/angiotensin II receptor blockers [ACEI/ARBs]), and laboratory measurements (serum albumin, hemoglobin, total cholesterol). Weight was measured without peritoneal dialysis fluid.

The primary outcome was all-cause mortality. Details for the CAPD follow-up were described in a study [16]. The follow-up period was from the start of PD to the date of death, transfer to hemodialysis, receiving renal transplantation, transfer to other dialysis centers, loss of follow-up, or May 31, 2023. Patients who were lost to follow-up were censored at the date of the last examination.

### Statistical analysis

Continuous variables were presented as means with standard deviations (SDs) for normally distributed data or medians with interquartile range (IQR) for skewed data. The normality of the parameters was examined using the Shapiro-Wilk test. Categorical variables were expressed as the number of patients. We first used restricted-cubic-spline plots to explore the non-linearity assumptions between TC/BA and the risk of all-cause mortality, fitting a restricted-cubic-spline function with four knots (at the 25th, 50th, 75th, and 95th percentiles) [17]. All patients were categorized based on the threshold value of TC/BA (hazard ratio [HR] = 1.0) using restricted cubic spline plots for the primary analysis. We defined malnutrition as TC/BA > = the threshold value.

Survival was calculated using the Kaplan-Meier method, and differences between survival distributions were assessed with a log-rank test. We primarily used cause-specific hazard models to explore the association of TC/BA with mortality risk. Patients who experienced the following events, such as transfer to hemodialysis, receiving renal transplantation, transfer to other centers, or loss of follow-up, were censored, which impeded the occurrence of death. Thus, transfer to hemodialysis, receiving renal transplantation, transferring to other centers, and losing follow-up were considered competing risks. We then constructed sub-distribution hazard models to confirm the association observed in the primary analysis. The main difference between the two hazard models is that subjects experiencing a competing risk event remain in the risk set in the sub-distribution hazard model but are removed in the cause-specific hazard model [18,19]. These models were constructed after adjusting the following variables: age, sex, current smoker, current alcohol use, comorbidities, medication use, and centers. The results from multivariable hazard models were presented as HRs and 95% confidence intervals (95% CIs). We conducted stratified analyses to assess potential effect modification by age (< 65 years, > = 65 years), sex, diabetes mellitus (yes or no), prior CVD (yes or no), and hypertension (yes or no).

To minimize the potential for reverse causation, we conducted analyses that excluded patients with prior CVD or those deaths in the first two years of follow-up. In addition, as for those patients with a short-term follow-up period, the interesting outcomes may only partially be observed with under-reporting of the incidence of mortality. We further analyzed the association in those patients with at least 24 months of follow-up for fully observing outcomes. All analyses were conducted with Stata 15.1. statistical software (StataCorp, College Station, TX).

## Results

### Baseline characteristics

**Figure 1.**
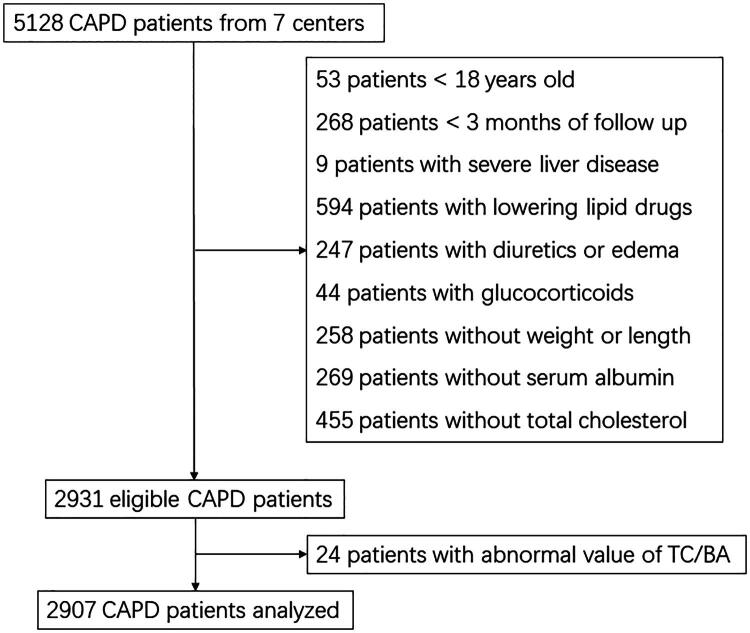
Flow chart. TC/BA, total cholesterol/body mass index × serum albumin.

Of 5128 patients from seven PD centers, we excluded 53 patients < 18 years old, 268 patients < 3 months of follow-up, nine patients with severe liver disease, 594 patients with lowering lipid drugs, 247 patients with diuretics or edema, and 44 patients with glucocorticoids. In addition, 982 patients without weight, height, serum albumin, or total cholesterol were also excluded (Figure 1). Thus, 2931 eligible patients were included, with 24 abnormal values of TC/BA. Finally, 2907 were analyzed in the present study. The baseline characteristics and demography were shown in Table 1. The median age was 50.0 (IQR 40.0–62.0) years old, and 1650 (56.8%) were male patients. 509 (17.5%) patients had DM, 1901 (65.4%) had hypertension, and 534 (18.4%) had a history of CVD. The median TC/BA levels were 2.23 (IQR 1.76–2.79), the median BMI, serum albumin, and total cholesterol values were 22.0 (IQR 20.0–24.2) kg/m^2^, 3.55 (IQR 3.17–3.90) g/dL, and 172.5 (142.1–205.0) mg/dL, respectively.

**Table 1.** Baseline characteristics categorized by TC/BA.

Variables	Total population	TC/BA < 2.24	TC/BA> =2.24	Standardize diff.	*P*-value
Number	2907	1457	1450		
TC/BA	2.23 (1.76–2.79)	1.76 (1.51–1.99)	2.79 (2.47–3.32)	2.40 (2.30, 2.49)	<0.001
Age, years	50.0 (40.0–62.0)	48.0 (39.0–59.0)	53.0 (41.0–64.0)	0.20 (0.12, 0.27)	<0.001
Sex, *n* (%)				0.35 (0.28, 0.43)	<0.001
Male	1650 (56.8%)	952 (65.3%)	698 (48.1%)		
Female	1257 (43.2%)	505 (34.7%)	752 (51.9%)		
BMI, kg/m^2^	22.0 (20.0–24.2)	23.1 (21.1–25.3)	20.9 (19.1–22.9)	0.31 (0.24, 0.39)	<0.001
Current smoker, *n* (%)	186 (6.4%)	115 (7.9%)	71 (4.9%)	0.12 (0.05, 0.20)	<0.001
Current alcohol use, *n* (%)	70 (2.4%)	41 (2.8%)	29 (2.0%)	0.06 (–0.01, 0.14)	0.217
Comorbidities					
DM, *n* (%)	509 (17.5%)	225 (15.4%)	284 (19.6%)	0.11 (0.04, 0.18)	0.003
Hypertension, *n* (%)	1901 (65.4%)	931 (63.9%)	970 (66.9%)	0.06 (–0.01, 0.14)	0.089
Prior CVD, *n* (%)	534 (18.4%)	218 (15.0%)	316 (21.8%)	0.18 (0.10, 0.25)	<0.001
Medication use					
CCB, *n* (%)	1942 (66.8%)	962 (66.0%)	980 (67.6%)	0.03 (–0.04, 0.11)	0.372
*β*-blockers, *n* (%)	1123 (38.6%)	565 (38.8%)	558 (38.5%)	0.01 (–0.07, 0.08)	0.87
*α*-blockers, *n* (%)	664 (22.8%)	325 (22.3%)	339 (23.4%)	0.03 (–0.05, 0.10)	0.491
ACEI/ARB, *n* (%)	1006 (34.6%)	453 (31.1%)	553 (38.1%)	0.15 (0.08, 0.22)	<0.001
Laboratory measurements					
Albumin, g/dL	3.55 (3.17–3.90)	3.70 (3.40–4.04)	3.35 (2.97–3.70)	0.47 (0.39, 0.54)	<0.001
Total cholesterol, mg/dL	172.5 (142.1–205.0)	146.2 (124.5–170.2)	200.3 (174.0–228.2)	1.48 (1.40, 1.56)	<0.001
Centers, *n* (%)				0.19 (0.12, 0.27)	<0.001
1	457 (15.7%)	206 (14.1%)	251 (17.3%)		
2	10 (0.3%)	4 (0.3%)	6 (0.4%)		
3	803 (27.6%)	404 (27.7%)	399 (27.5%)		
4	322 (11.1%)	142 (9.8%)	180 (12.4%)		
5	55 (1.9%)	19 (1.3%)	36 (2.5%)		
6	838 (28.8%)	470 (32.3%)	368 (25.4%)		
7	422 (14.5%)	212 (14.6%)	210 (14.5%)		

TC/BA, total cholesterol/ body mass index*serum albumin; DM, diabetes mellitus; CVD, cardiovascular disease; CCB, calcium channel blocker; ACEI/ARB, angiotensin-converting enzyme inhibitors/angiotensin II receptor blockers.

**Figure 2.**
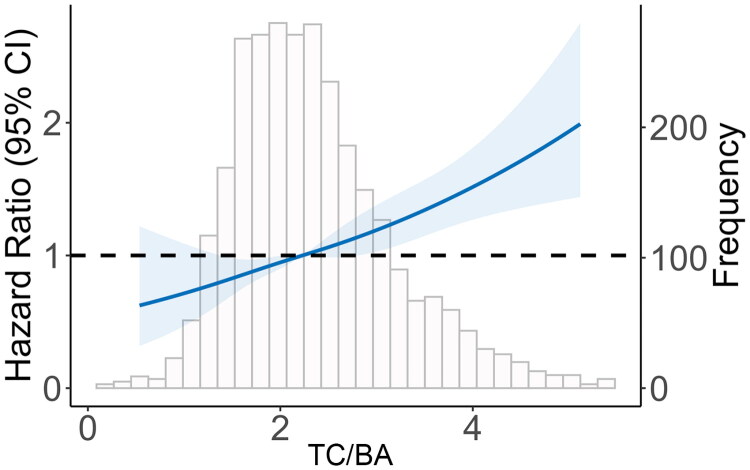
Association of TC/BA with risk of mortality. The plot was adjusted for age, sex, current smoker, current alcohol use, comorbidities, medication use, and centers. Dashed lines indicate 95% confidence intervals. The median TC/BA (2.24) was the reference standard, indicated by the grayline. TC/BA, total cholesterol/body mass index × serum albumin.

As a positive linear association (nonlinear, *p* = 0.374) were observed from restricted cubic spline plots (Figure 2), we found the TC/BA value 2.24 (HR = 1.0) as the threshold value for all-cause mortality. All patients were categorized into two groups: the low group (TC/BA < 2.24) and the high group (TC/BA > = 2.24). In the present study, we defined the TC/BA value > = 2.24 as malnutrition in CAPD patients, and the prevalence was 49.9%. Table 1 showed the baseline demography and characteristics of the two groups. Compared with those in the low group, patients in the high group had older age, more frequency of females, DM, prior CVD, and using ACEI/ARB. In addition, a significant difference in proportions was observed in the two groups from the sixth center. Notably, the high group had lower BMI and serum albumin but had higher total cholesterol.

Baseline demography and characteristics were well balanced between the total population and those excluded patients without BMI, serum albumin, or total cholesterol (data not shown).

### TC/BA and all-cause mortality

During the 12469.4 person-years of follow-up, 754 (25.9%) patients died, 282 (9.7%) patients transferred to hemodialysis, 153 (5.3%) patients received renal transplantation, 24 (0.8%) patients transferred to other dialysis centers, and 54 (1.9%) patients had been the loss of follow-up. Of 754 deaths, 351 (46.6%) deaths were due to CVD, 115 (15.3%) deaths due to infectious disease, 27 (3.6%) deaths due to gastrointestinal bleeding, 21 (2.8%) deaths due to malignancy, 96 (12.7%) deaths due to other reasons, and 94 (12.5%) deaths due to unknown reasons. In addition, of 754 deaths, 287 (38.1%) were in the low group and 467 (61.9%) in the high group. All-cause mortality incidence was 60.5/1000, 47.2/1000, and 73.1/1000 patient-year in the total population, the low and the high groups, respectively.

The high group had a significantly higher cumulative risk of death than the low group (Figure S1). In the multivariate cause-specific hazard model, a per-1.0 increase of TC/BA had a 1.29-fold (95% CI 1.19–1.39) risk of all-cause mortality in the total population after adjustments for demographic factors, comorbidities, medication use, and centers (the multivariate model in Table 2). Compared with the low group, the high group had a 1.29-fold (95% CI 1.11–1.50) risk of all-cause mortality after adjusting for confounding factors (Table 2).

**Table 2.** Association between TC/BA (continuous) and all-cause mortality using cause-specific hazard models*.

	Death/number	HR (95% CI)
Univariate model	754/2907	1.46 (1.35 to 1.57)
Multivariable model	754/2907	1.29 (1.19 to 1.39)
Patients without prior cardiovascular disease	497/2373	1.35 (1.23 to 1.49)
Patients without deaths in the first two years of follow-up	518/2671	1.25 (1.13 to 1.38)
Patients with a follow-up period > = 24 months	518/2268	1.29 (1.17 to 1.42)

*Per-1.0 increase of TC/BA; Unless stated, model adjusted for age, sex, current smoker, current alcohol use, comorbidities, medication use, and centers. TC/BA, total cholesterol/ body mass index*serum albumin; HR, hazards ratio; CI, confidence interval.

We confirmed this association using a sub-distribution hazard model. When using sub-distribution hazard models, a pre-1.0 increase in TC/BA had a 1.14-fold (95% CI 1.07–1.21) risk of all-cause mortality (Table S1). Also, compared with the low group, the high group had a 1.21-fold (95% CI 1.10–1.45) risk of all-cause mortality after adjusting for confounding factors (Table S2).

### Sensitivity analysis

We performed sensitivity analyses in patients without prior CVD, without deaths during the first two years of follow-up, or the follow-up period > = 24 months. Similar results were observed in three specific populations (Tables 2 and 3). Notably, HR of TC/BA (per-1.0 increase) was 1.32 (95% CI 1.23–1.41), 1.29 (95% CI 1.19–1.41), and 1.25 (95% CI 1.22–1.27) times higher than the HR of BMI (per-1.0 decrease), total cholesterol (per-10 increase), and serum albumin (per-1.0 decrease), respectively.

**Table 3.** Association between TC/BA (categories) and all-cause mortality using cause-specific hazard models*.

	Death/number	HR (95% CI)	
		Low group (< 2.24)	High group(≥ 2.24)
Univariate model	754/2907	1.0	1.53 (1.32 to 1.77)
Multivariable model	754/2907	1.0	1.29 (1.11 to 1.50)
Patients without prior cardiovascular disease	497/2373	1.0	1.37 (1.14 to 1.65)
Patients without deaths in the first two years of follow-up	518/2671	1.0	1.27 (1.06 to 1.53)
Patients with a follow-up period > = 24 months	518/2268	1.0	1.28 (1.07 to 1.53)

*Unless stated, the model adjusted for age, sex, current smoker, current alcohol use, comorbidities, medication use, and centers. TC/BA, total cholesterol/ body mass index*serum albumin; HR, hazards ratio; CI, confidence interval.

### Subgroup analyses

Associations of TC/BA with all-cause mortality in subgroups were shown in Table 4. Similar results were observed in all subgroups. There were no significant subgroup interactions. Restricted cubic spline plots for all-cause mortality showed nearly positive linear relationships between TC/BA and all-cause mortality, observed in all subgroups except those with DM (Figure S2). Notably, when using serum albumin < 3.8 g/dL or total cholesterol < 100 mg/dL to define malnutrition, malnutrition prevalences were 70.2 and 3.8%, respectively.

**Table 4.** Association between TC/BA (categories) and all-cause mortality in subgroups*.

Subgroups	Death/number	HR (95% CI)	P for interaction
Age > = 65 years old	290/560	1.24 (1.09 to 1.41)	0.515
Age < 65 years old	464/2347	1.35 (1.21 to 1.49)	
Male	406/1650	1.25 (1.11 to 1.40)	0.579
Female	348/1257	1.32 (1.17 to 1.48)	
DM	235/509	1.25 (1.09 to 1.43)	0.995
Without DM	519/2398	1.32 (1.19 to 1.46)	
Hypertension	581/1901	1.28 (1.17 to 1.40)	0.792
Without hypertension	173/1006	1.34 (1.12 to 1.62)	
Prior CVD	257/534	1.22 (1.05 to 2.19)	0.156
Without a history of CVD	497/2373	1.33 (1.21 to 1.47)	

*Per 1.0 increase of TC/BA; Unless stated, the model adjusted for age, sex, current smoker, current alcohol use, comorbidities, medication use, and centers. TC/BA, total cholesterol/ body mass index × serum albumin; HR, hazards ratio; CI, confidence interval.

## Discussion

In the retrospective cohort study, we first reported that TC/BA had a positive linear relationship with all-cause mortality in CAPD patients, suggesting patients with higher TC/BA had higher risks of all-cause mortality in these patients. Our findings were robust because we showed consistent results across different hazard models, several sensitivity analyses, and subgroup analyses. Notably, when using serum albumin or total cholesterol to define malnutrition, prevalences of malnutrition were significantly different (70.2 vs. 3.8%). These findings prompted us to combine indicators to assess nutritional status. Notably, the HR of TC/BA was significantly higher than the HR of BMI, total cholesterol, and serum albumin, respectively. In addition, when using serum albumin < 3.8 g/dL or total cholesterol < 100 mg/dL to define malnutrition, malnutrition prevalences were 70.2 and 3.8%, respectively. Based on these findings above, we draw a conclusion that TC/BA may outperform each index in assessing these patients’ prognosis. In clinical practice, the combined nutritional index may more comprehensively and favorably predict these patients’ prognosis.

Although much progress has been made in improving the nutritional status of patients, the prevalence of PEW in dialysis patients remains high, ranging from 18 to 56%, depending on the assessment method used [20–23]. Several recognized methods exist for stratifying the malnutrition status of peritoneal dialysis patients. One indicator used to assess nutritional status is serum albumin. This indicator is helpful because its level is influenced by daily protein intake [24–26]. In addition, serum albumin is low-cost and readily available [27]. However, albumin as a diagnostic indicator is limited because albumin levels may be affected by various factors, such as liver pathology and gastrointestinal and renal losses [28]. Individuals who start peritoneal dialysis with nephrotic-range proteinuria and significant urine output may have low serum albumin but have no other evidence of malnutrition. It has been shown that the average rate of albumin synthesis is approximately 12 g/day, corresponding to a fractional albumin catabolic rate of approximately 4%/day (assuming total body albumin is approximately 270 grams in a normal individual), one-half of which is extravascular [29]. In patients on peritoneal dialysis with chronic inflammation, there tends to be a decrease in overall albumin synthesis [30]. Finally, volume expansion may be associated with hypoalbuminemia. Hence, it is unclear whether all individuals with biochemical abnormalities, such as low serum albumin, are truly malnourished. In addition, BMI is the most commonly used weight-for-height measure among body mass indicators. It may be used to assess PEW [31,32]. However, BMI can be heavily influenced by fat mass or hydration status. The ISRNM panel recommends that a BMI less than 23 kg/m^2^ is a marker of PEW [9]. The panel also recognizes that the threshold for this criterion may need further adjustment, especially in some populations, such as those from Southeast Asia, where a low BMI may not indicate pathology [33,34]. In the present study, all patients were from Southeast Asia. Total cholesterol is always influenced by lowering lipid drugs. Thus, more than biochemical parameters alone may be required to diagnose malnutrition. when using plasma albumin to assess a patient’s nutritional status, we must incorporate other nutritional indicators. A valuable tool for a comprehensive assessment of nutritional status is the subjective global assessment (SGA). Parameters analyzed include medical history and physical examination [35]. The former considers dietary intake, weight change, gastrointestinal complaints, functional capacity, and nutritional requirements in the medical history. In contrast, the physical examination findings focus on signs of adiposity, muscle atrophy, and altered fluid balance due to undernutrition [35]. The Malnutrition-Inflammation Score (MIS) is a modified version of the SGA. Other parameters of this assessment tool include serum albumin levels, total iron binding capacity, and body mass index [36]. KDOQI recommends using the MIS to assess nutritional status rather than the SGA [37]. It is also difficult to accurately assess a patient’s nutritional status with these indicators, including dietary intake and gastrointestinal complaints. Bioimpedance analysis (BIA) is a method for researchers to measure lean and fat body mass and intra- and extracellular fluid volumes. It is essential for assessing hydration status [38]. A study reported that in addition to managing dry body mass, BIA can be used to assess the nutritional status of patients with PD [39]. However, this test is not widely available in primary health centers. In the present study, we combined these three indicators, i.e. BMI, serum albumin, and total cholesterol, for the first time to assess the nutritional status of patients and further explored the relationship between combined indicators and patients’ mortality. We found a positive linear relationship between the combined indicator and mortality. The combined nutritional indicator was not only easy to manipulate in clinical practice but also can be an excellent way to assess the prognosis of patients. A recent study found that albumin to total cholesterol ratio before the start of PD between 0.77 and 0.82 was associated with a lower risk of death than a higher or lower ratio, resulting in a U-curve association [14]. However, we need a more straightforward and easier-to-implement nutritional indicator for assessing patient prognosis. Fortunately, the combined nutritional indicator met the criteria above. Our findings further improve nutritional metrics to assess patient prognosis. Nonetheless, our findings need to be verified for accuracy and reliability in future clinical practice. The detailed mechanism remained to be explored. Nonetheless, a simple relationship between TC/BA and mortality makes it easier to stratify the clinical prognosis of these patients.

The strengths of our study included a large number of patients, strict screening criteria, and rigorous different multivariate hazard models. Nevertheless, some limitations should be mentioned. First, the ISRNM panel recommends that a BMI less than 23 kg/m^2^ is a marker of PEW [9]. However, those from Southeast Asia with a low BMI may not indicate pathology [32,33]. Although we excluded patients without edema or diuretics, a lower BMI might be desirable for specific Asian populations [9]. We did not do the subgroup analysis because all patients were from Southeast Asia [9]. Second, we analyzed only TC/BA values at the onset of PD. The single TC/BA measurement at baseline may have underestimated this association because of regression dilution bias [40]. Regression dilution bias may have contributed to some extent to the overestimation of this association [41]. Nonetheless, serial measurements may provide more information than single measurements. Third, we did not include prealbumin in the composite nutritional index. However, prealbumin was not recommended as a nutritional parameter of the same usefulness as serum albumin [42]. Fourth, we had excluded patients with severe liver pathology and gastrointestinal disease, which leaded to losses of serum albumin. However, residual kidney function in PD patients may lead to renal losses of serum albumin, which may affect the association between TC/BA and mortality. We had added these sentences to the limitation section. Finally, all eligible patients were from China, suggesting that the findings may not apply to other ethnic groups. In addition, automated PD may result in a significant decrease in serum albumin and an increase in total cholesterol levels [43,44]. Therefore, our results are also not applicable to those using automated cyclers.

In conclusion, TC/BA had a positive linear relationship with all-cause mortality in CAPD patients. More importantly, the panel of nutritional indexes may outperform each index and more comprehensively and favorably assess the prognosis in these patients. Our findings were helpful to quickly and reliably evaluate the relationship between nutritional status and prognosis. The combined nutritional index may further improve mortality risk stratification in CAPD patients. Nonetheless, our findings need to be validated in future prospective studies with large sample sizes.

## Supplementary Material

Supplementary materials.docx
