# Supplementary material for: A combined nutritional index and mortality in patients with peritoneal dialysis
Source: Ren Fail. 2025 Aug 10;47(1):2541069. doi: 10.1080/0886022X.2025.2541069 (PMC12340938; doi:10.1080/0886022X.2025.2541069)
Supplement: Supplementary materials.docx [file IRNF_A_2541069_SM5041.docx]

**Supplementary materials**

A combined nutritional indicator and mortality in patients with peritoneal dialysis

**Table S1. Association between TC/BA (continuous) and all-cause mortality** **using** **sub-distribution hazard models***

|  | HR (95% CI) |
| --- | --- |
| Univariate model | 1.15 (1.06 to 1.24) |
| Multivariable model | 1.14 (1.07 to 1.21) |
| Patients without prior cardiovascular disease | 1.17 (1.09 to 1.26) |
| Patients without deaths in the first two years of follow-up | 1.15 (1.07 to 1.24) |
| Patients with a follow-up period >= 24 months | 1.14 (1.06 to 1.22) |

*Per-1.0 increase of TC/BA; Unless stated, the model adjusted for age, sex, current smoker, current alcohol use, comorbidities, medication use, and centers. TC/BA, total cholesterol/ body mass index*serum albumin; HR, hazards ratio; CI, confidence interval.

**Table S2. Association between TC/BA (categories) and all-cause mortality** **using sub-distribution hazard models****

|  | HR (95% CI) | |
| --- | --- | --- |
|  | Low group (< 2.24) | High group(≥ 2.24) |
| Univariate model | 1.0 | 1.43 (1.25 to 1.57) |
| Multivariable model | 1.0 | 1.21 (1.10 to 1.45) |
| Patients without prior cardiovascular disease | 1.0 | 1.20 (1.08 to 1.59) |
| Patients without deaths in the first two years of follow-up | 1.0 | 1.11 (1.02 to 1.44) |
| Patients with a follow-up period >= 24 months | 1.0 | 1.19 (1.03 to 1.45) |

*Unless stated, the model adjusted for age, sex, current smoker, current alcohol use, comorbidities, medication use, and centers. TC/BA, total cholesterol/ body mass index*serum albumin; HR, hazards ratio; CI, confidence interval.


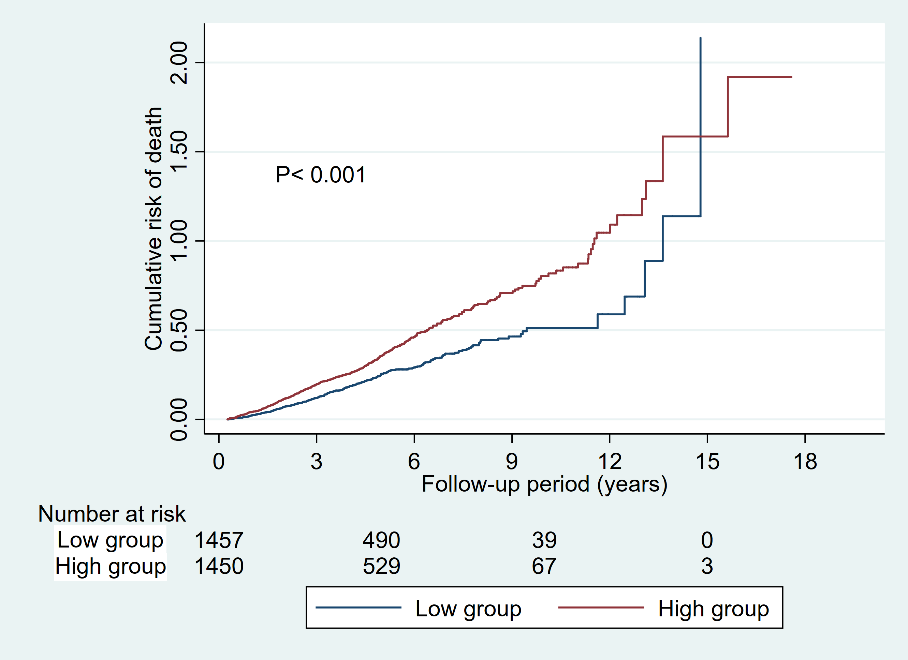


**Figure S1. Cumulative mortality by** **categories of TC/BA.**

TC/BA, total cholesterol/ body mass index*serum albumin.


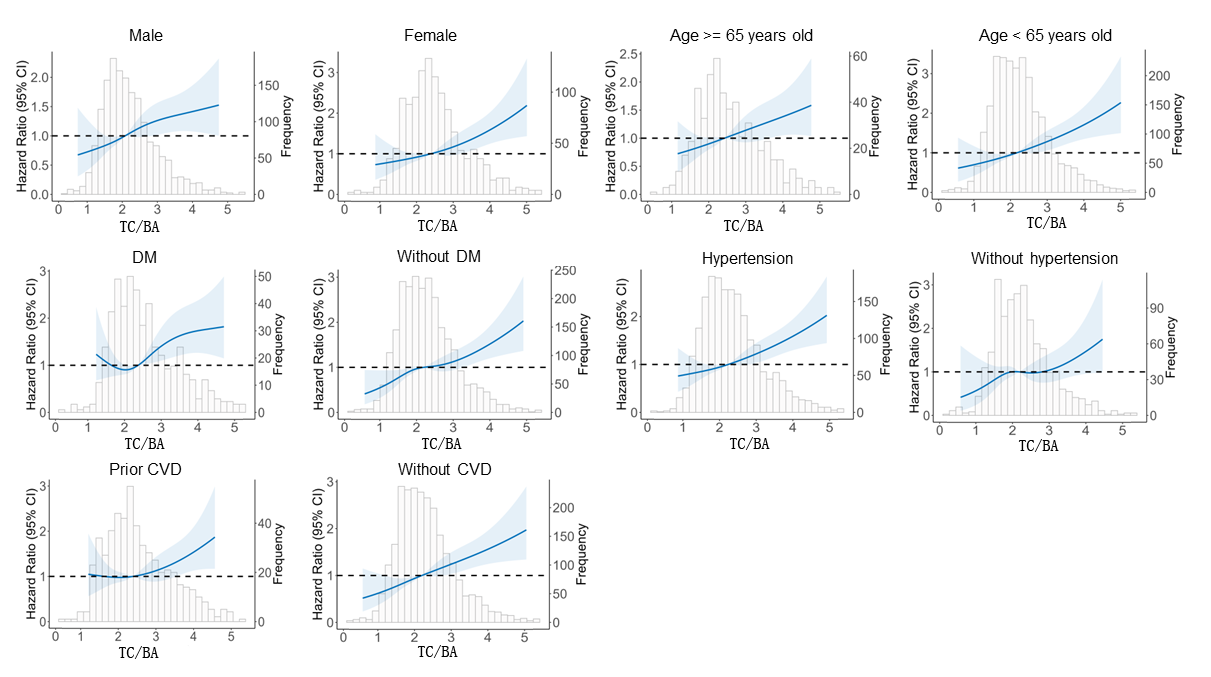


**Figure S2. Association of TC/BA with risk of mortality in subgroups.**

The plots were adjusted for age, sex, current smoker, current alcohol use, comorbidities, medication use, and centers in subgroups except for the subgroup variable. Dashed lines indicate 95% confidence intervals. The median TC/BA (2.24) was the reference standard, indicated by the grayline.

TC/BA, total cholesterol/ body mass index*serum albumin.
